# Novel Glycidyl Carbamate Functional Epoxy Resin Using Hydroxyl-Terminated Polybutadiene

**DOI:** 10.3390/polym16223107

**Published:** 2024-11-05

**Authors:** Hae-Chan Kim, Sun-Jae Moon, Yong-Rok Kwon, Seok-kyu Moon, Dah-hee Kim, Dong-Hyun Kim

**Affiliations:** 1 User Convenience Technology R&D Department, Korea Institute of Industrial Technology (KITECH), Ansan-si 15588, Republic of Korea; coolskawk@kitech.re.kr (H.-C.K.); sweet8320@kitech.re.kr (S.-J.M.); yongrok@kitech.re.kr (Y.-R.K.); anstjrrb@kitech.re.kr (S.-k.M.); dahhee@kitech.re.kr (D.-h.K.); 2 Department of Materials Science and Chemical Engineering, Hanyang University, Ansan-si 15588, Republic of Korea

**Keywords:** hydroxyl-terminated polybutadiene, glycidyl carbamate, epoxy resin, dual-cure adhesive

## Abstract

Herein, a novel glycidyl carbamate functional epoxy resin (GCE) is synthesized by the additional reaction of the isocyanate group of tolylene diisocyanate (TDI) with the hydroxyl group of hydroxyl-terminated polybutadiene (HTPB) and glycidol. The successful synthesis of the GCE is confirmed by FT-IR and ^1^H NMR spectroscopy. Furthermore, a dual-curing adhesive system is developed using acrylic acid and trimethylolpropane triacrylate with varying GCE contents, and its adhesive performance is assessed by testing adhesive strength, pencil hardness, and surface energy. As a result, the dual-cure adhesive containing 0.2 mol of GCE demonstrates an impressive adhesive strength of 11.1 MPa, a pencil hardness of B, and surface energy comparable to that of standard polycarbonate film.

## 1. Introduction

Thermosetting polymers are integral to a wide range of applications that necessitate exceptional performance, environmental resilience, and prolonged durability [1]. These polymers are synthesized through the polymerization of monomers or resins that contain multiple functional groups. During the curing process, these functional groups react to initiate gelation, culminating in the formation of highly crosslinked three-dimensional networks [1,2]. In modern applications, epoxy and polyurethane technologies stand out as two of the most extensively used thermosetting systems. Epoxies are valued for their strong adhesion and corrosion resistance, making them ideal for chemical protection. Polyurethanes, especially aliphatic variants, are often used as topcoats due to their durability and outdoor resilience, particularly against UV exposure. Together, these systems offer complementary strengths, with epoxies excelling in protection and adhesion, and polyurethanes providing mechanical toughness and weather resistance [3].

Epoxy resins are compounds that have multiple epoxy groups and can be cured through a variety of chemistries. The most common epoxy resin family is based on the diglycidyl ether of bisphenol A [4]. A range of molecular weights can be synthesized, leading to resins that are either low-viscosity liquids or solids. Glycidyl ethers of a number of other compounds, including various diols and phenolic resins, are also commercially available [2,4]. For instance, the glycidyl carbamate functional group consists of an epoxide adjacent to a carbamate or urethane. Glycidyl carbamate functional resins have two or more glycidyl carbamate groups [5] and can be cured via these epoxy groups with any typical curing agent, such as an amine, anhydride, or phenolic. An advantage of the glycidyl carbamate resin is that the end user can use epoxy curing chemistry to form thermosets and obtain a polyurethane without having to handle any isocyanates. Glycidyl carbamate compositions have been explored for use in adhesives [6,7], sealants, elastomers, and coatings [8]. In most of the reported studies, glycidyl carbamate resins offer improved adhesion, flexibility, and faster curing compared to conventional epoxy resin systems, making them an attractive alternative thermosetting polymer system [5,7].

Meanwhile, polyurethanes are produced by the reaction of a polyisocyanate and a polyol, with both aromatic and aliphatic polyisocyanates being commonly used. Additionally, various oligomers and adducts of polyisocyanates are available to modify the properties of the final product [9]. Due to the rapid reaction between isocyanates and polyols, a multifunctional isocyanate is typically mixed with the polyol just prior to application, allowing for curing to occur directly on the substrate. Among the polyols, hydroxyl-terminated polybutadiene (HTPB), a telechelic liquid rubber, is particularly notable [10,11]. HTPB is produced through the hydrogen peroxide-initiated polymerization of butadiene and is widely used in the formulation of elastomers and adhesives due to its flexible properties [12,13].

In this present study, an epoxy resin was synthesized using HTPB through the introduction of a glycidyl carbamate functional group, allowing the resin to retain the inherent properties of HTPB while functioning effectively as an epoxy resin. This modification enables the combination of HTPB’s unique flexibility and heat resistance with the crosslinking characteristics of epoxy resins, making it suitable for a wide range of high-performance applications, including advanced adhesives for electronics, protective coatings for automotive components, and materials used in aerospace engineering that demand both thermal stability and mechanical durability. The characteristics of the novel glycidyl carbamate functional epoxy resin (GCE) are identified using an ultraviolet (UV)/thermal dual-cure adhesive (DCA) system. Here, the UV irradiation induces a binding reaction of the GCE with the C=C double bond of the HTPB acrylate group. Then, by sequential thermosetting, the epoxy groups at each end of GCE induce a ring-opening reaction. The improved adhesion characteristics of the as-synthesized glycidyl carbonate functional group are also demonstrated herein. In addition, the intrinsic and excellent heat resistance of the HTPB is reinforced by the inclusion of molecular ring structures of tolylene diisocyanate (TDI). The results of this study are expected to provide key insights for the design and development of new epoxy resins, particularly for use in industries that require enhanced thermal stability, mechanical strength, and superior adhesive properties, such as aerospace engineering, high-performance electronics, and automotive manufacturing.

## 2. Materials and Methods

### 2.1. Materials

For the synthesis of GCE, hydroxyl-terminated polybutadiene (HTPB; Evertech Enterprise Co., Ltd., Hwaseong, Republic of Korea), tolylene-2,4-diisocyanate (TDI; 95%, Sigma Aldrich, St. Louis, MO, USA), and (±)-glycidol (Sigma-Aldrich, St. Louis, MO, USA) were used as reactants. Dibutyltin dilaurate (DBTDL; 95%, Sigma-Aldrich, St. Louis, MO, USA) served as the catalyst, 2-butanone (95%, Samchun Chemicals, Seoul, Republic of Korea) was used as the solvent, and hydroquinone (≥99%, Sigma-Aldrich, St. Louis, MO, USA) acted as an inhibitor. Acrylic acid (AA; 99%, Sigma-Aldrich, USA), trimethylolpropane triacrylate (TMPTA; Sigma-Aldrich, St. Louis, MO, USA), and bisphenol A diglycidyl ether (BADGE; Sigma-Aldrich, St. Louis, MO, USA) were utilized as a comonomer and crosslinker, respectively, without further purification. The photoinitiator 2,2-dimethoxy-2-phenylacetophenone (DMPA; Sigma-Aldrich, St. Louis, MO, USA) and the thermal curing agent 1-methylimidazole (1-MI; ≥99%, Sigma-Aldrich, St. Louis, MO, USA) were also used without purification. For the adhesion test, polycarbonate (PC) film (1T, Hwa-in Science, Seoul, Republic of Korea) was employed as purchased.

### 2.2. Glycidyl Carbamate Functional Epoxy Resin (GCE)

**Figure 1:**
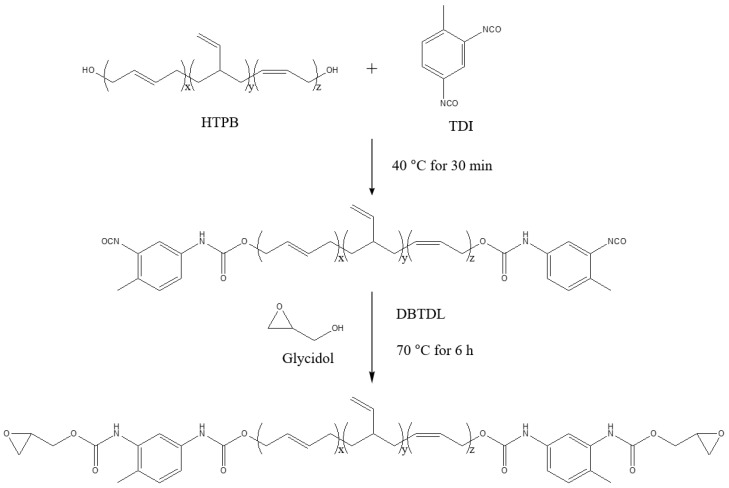
The schematic synthesis of GCE from HTPB, TDI, and glycidol.

The GCE, modified at both ends of HTPB, was synthesized using a 2:1 equivalent ratio of TDI to HTPB (with an NCO ratio of 1:1). Subsequently, glycidol was introduced in a 1:1 ratio with TDI to complete the final reaction. The reactants and reaction conditions are illustrated schematically in Figure 1.

The reactions took place in a 500 mL four-neck flask fitted with a mechanical stirrer, reflux condenser, thermometer, and a nitrogen gas injection needle. In each reaction, HTPB (0.2 mol) and TDI (0.4 mol) were stirred in 30 mL of 2-butanone at 250 rpm. The monomers were vigorously mixed at 40 °C for 30 min, after which the temperature was gradually increased to 70 °C. At this point, DBTDL (0.01 wt.%) and hydroquinone (200 ppm) were added, and the mixture was kept at 70 °C. The reaction was tracked by monitoring the NCO peaks with FT-IR spectroscopy and was terminated when the NCO peak was no longer observed. After the reaction, the mixture was allowed to cool and kept at room temperature for one hour. It was then washed three times with deionized water to remove any remaining unreacted monomers and by-products. As a result, a transparent, high-viscosity GCE was obtained.

### 2.3. Preparation of the Dual-Cure Adhesive

AA, TMPTA, and GCE were combined in predetermined amounts and stirred until a uniform mixture was obtained. Afterward, DMPA (0.1 phr) and 1-MI (0.25 phr) were incorporated, followed by further stirring and degassing through sonication. The degassed mixture underwent UV curing at 360 nm with a dose of 300 mJ/cm^2^ for a few seconds, followed by thermal curing in a convection oven at 90 °C for 30 min. The compositions and designations of the different DCA samples are listed in Table 1. In addition, the commonly used BADGE was set as a control group in the evaluation of several properties. The DCA_BADGE_2_ was manufactured with 0.2 mol of BADGE. All samples were cured under the same conditions. To minimize chaotic effects, the adhesive network components were kept simple and consistent; i.e., no fillers or reinforcing agents were added.

### 2.4. Structural Analysis

The structure of the GCE in the form of a potassium bromide pellet was analyzed using Fourier transform infrared (FT-IR) spectroscopy (NEXUS Instruments; Thermo Nicolet NEXUS 670, Thermo Fisher Scientific, Waltham, MA, USA) in the range of 600–4000 cm^−1^ with a resolution of 1 cm^−1^ and further examined via nuclear magnetic resonance (^1^H NMR) spectroscopy (Varian; Unity Inova 500 MHz, Varian Inc., Palo Alto, CA, USA).

### 2.5. Thermogravimetric Analysis (TGA)

Thermal stability is a critical property of thermosetting resins. Therefore, the thermal stability and decomposition behavior of the samples were assessed using the widely applied thermogravimetric analysis (TGA), which tracks weight loss as a function of temperature [14]. The TGA analysis was conducted under a nitrogen atmosphere, with heating from 30 to 800 °C at a rate of 20 °C/min, and the weight loss of the samples was recorded as a function of temperature.

### 2.6. Gel Fraction

The gel fractions of the DCAs (5 g) were measured after UV/thermal curing. The cured DCAs were determined by soaking in methyl ethyl ketone (500 mL) for 24 h. The insoluble part was removed by filtration and dried at 60 °C to a constant weight. The gel fraction was then calculated using Equation (1):

(1) $$Gel\;fraction=\frac{\omega_{e}}{\omega_{i}}$$

where *ω*_i_ and *ω*_e_ are the weights of the initial and extracted dried DCA, respectively.

### 2.7. Adhesive Strength

**Figure 2:**
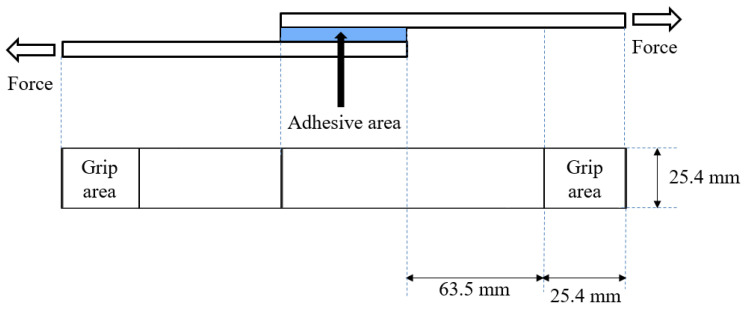
A schematic diagram of the lap shear strength measurement.

Lap shear strength is a commonly used method to assess adhesive strength. In this study, the lap shear strengths of the dual-cure specimens were evaluated following the ASTM D 1002 standard, using a universal testing machine (AllroundLine Fmax 10 kN UTM; ZwickRoell GmbH & Co. KG, Ulm, Germany), as illustrated schematically in Figure 2. Specimens were prepared on polycarbonate (PC) films with adhesion areas of 25.4 mm × 5 mm and an average thickness of 40 µm for this testing procedure.

### 2.8. Pencil Hardness

Pencil hardness testing is commonly used in industry to assess the surface properties of coatings, although the hardness values obtained are less precise compared to those from micro-hardness tests [15]. In this study, the pencil hardness was evaluated using a pencil hardness tester (HT-6510P; LANDTEK, Guangzhou, China) with a 1000 g load. During testing, the pencil (6B–6H, ChungHwa, Shanghai, China) was mounted on the device at a 45° angle to the test surface.

### 2.9. Surface Energy

Measuring surface energy is essential for confirming compatibility with other materials. In this study, surface energy was determined using a contact angle analyzer (Phoenix-MT(T); Surface Electro Optics Co., Suwon-si, Republic of Korea) via the sessile drop method. For each sample, the contact angle was measured five times at room temperature, and surface energy was calculated by analyzing the contact angles of water and ethylene glycol using Surfaceware 9.0 software.

## 3. Results and Discussion

### 3.1. Characterization of the GCE

**Figure 3:**
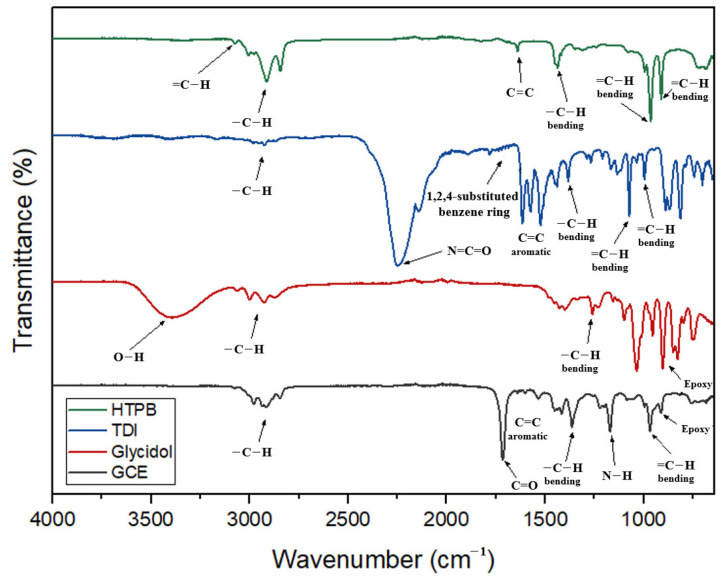
The FT-IR spectra of the starting materials (HTPB, TDI, and glycidol) and the product GCE.

The FT-IR spectra of the starting materials (HTPB, TDI, and glycidol) are compared with those of the product (GCE) in Figure 3. Here, the strong peak at 2269 cm^−1^ in the TDI spectrum (blue line) due to the NCO stretching vibrations [16], and the broad absorption band at 3341 cm^−1^ in the glycidol spectrum (red line) attributed to the stretching vibrations of the hydroxyl (O−H) group, are completely absent in the final GCE product (black line). Meanwhile, a peak is observed at 930 cm^−1^ in the GCE spectrum due to the reduction of the epoxy group [17], along with an absorption band at about 1570−1630 cm^−1^ due to the in-plane vibration of the C=C bonds of the aromatic ring derived from TDI [18]. In addition, peaks due to the C=O and N−H stretching vibrations of the urethane bonds are observed at 1716 cm^−1^ and 1170 cm^−1^, respectively [19].

**Figure 4:**
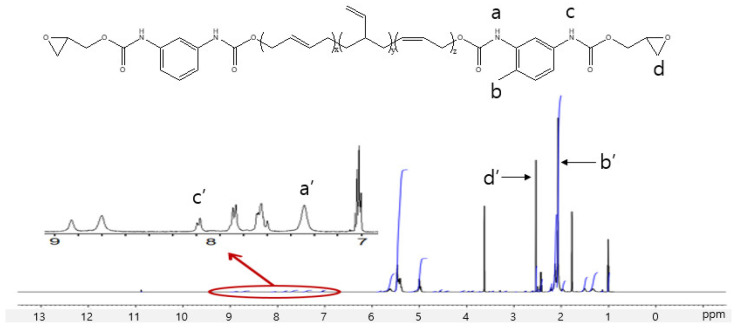
The ^1^H NMR spectrum of the GCE (a, a’: the allophanate NH of the urethane bonds; b, b’: the aromatic methyl protons of the urethane, allophanate, and unreacted TDI; c, c’: the NH of the urethane bonds of para position to the methyl group; d, d’: the epoxy group).

The ^1^H NMR spectrum of the GCE is presented in Figure 4. Here, the peaks at 2.10 to 2.25 ppm are attributed to the aromatic methyl protons of the urethane, allophanate, and unreacted TDI, while the peaks between 7.00 and 8.66 ppm are attributed to the aromatic protons. The integral of this area is nearly equivalent to that of the methyl protons. Additionally, the characteristic peaks corresponding to the allophanate NH in the urethane bonds are observed at 7.68 ppm (stemming from the lower reactivity of the NCO group in the ortho position to the methyl group) and 8.07 ppm (arising from the higher reactivity of the NCO group located in the para position relative to the methyl group) [20].

### 3.2. Curing Characteristics

**Figure 5:**
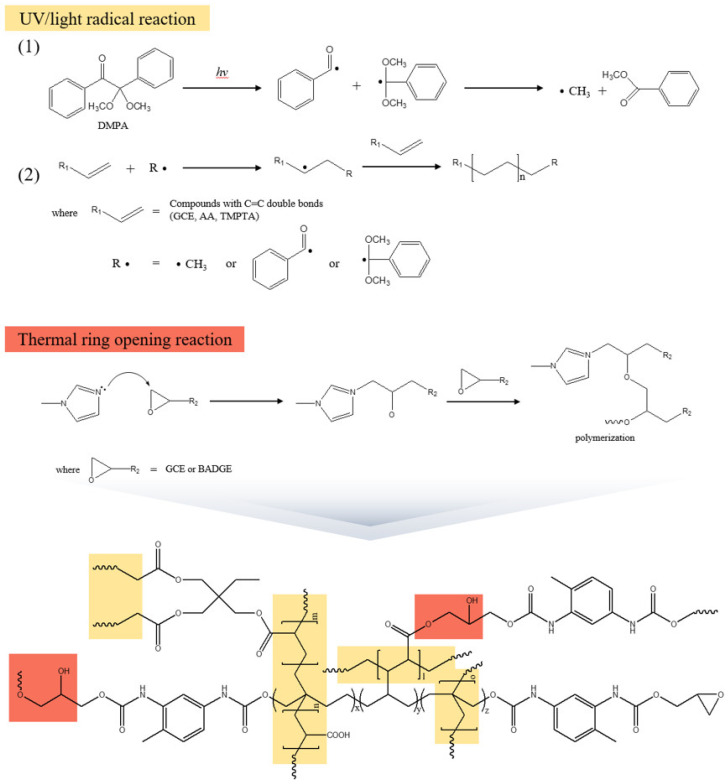
A schematic diagram of the dual-cured adhesive network with GCE.

Figure 5 shows a schematic diagram of the proposed network configuration formed by the dual curing (UV/thermal) of the GCE-containing adhesive system. In this process, the UV initiation triggers the reaction between GCE, AA, and TMPTA, which is propagated by the decomposition and radical activity of DMPA. Subsequently, a carboxyl group derived from AA is bonded to an ester through the ring-opening reaction of an epoxy group of GCE under the catalyst of imidazole by sequential thermal initiation. In addition, hydrogen bonds can occur between each grown chain or intermolecular of nitrogen atoms in urethane bonds.

**Figure 6:**
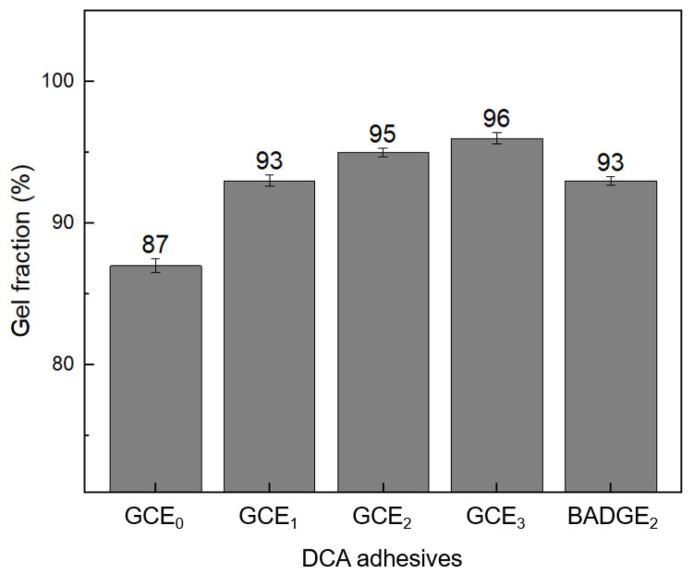
The gel fractions of the DCA adhesives.

Observing gel fractions provides an effective and reliable method for assessing the insoluble portions of polymers, particularly those within crosslinked or networked structures. The gel fractions of DCA samples, thermally cured at 90 °C for 30 min following UV curing, are presented in Figure 6. All DCA samples containing GCE exhibited a gel fraction of 92% or higher. The progressive increase in gel fraction with higher GCE content can be explained by the greater number of crosslinking sites available due to the molecular structure of GCE. As the GCE content increases, the availability of C=C radicals rises, facilitating more extensive UV-induced crosslinking during the curing process. This results in a denser polymer network with fewer soluble components, as indicated by the higher gel fraction. In comparison, the gel fraction for the BADGE-based adhesive is slightly lower than that of GCE_2_ and GCE_3_. This can be attributed to the structural characteristics of BADGE, which forms a more linear and less flexible network. The lower crosslinking density of BADGE limits the extent of the network formation, resulting in a slightly lower gel fraction. Additionally, the presence of the glycidyl carbamate group in GCE likely contributes to enhanced crosslinking efficiency, leading to higher gel fractions in the GCE-based samples.

### 3.3. Adhesive Properties

**Figure 7:**
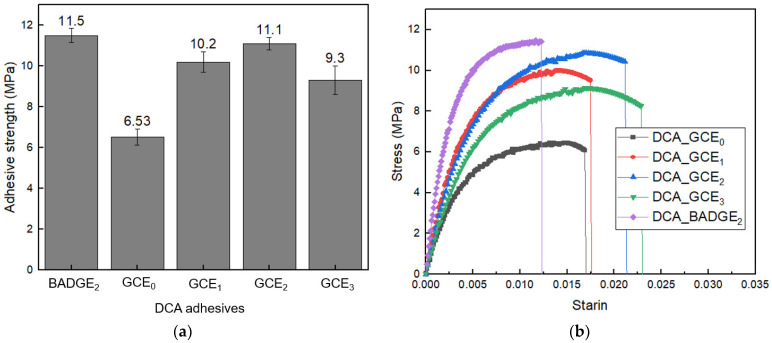
The adhesive strengths (**a**) and stress–strain curve (**b**) of the DCA_GCE adhesives and DCA_BADGE_2_.

The adhesive strengths of the DCA_GCE_0_, DCA_GCE_1_, DCA_GCE_2_, and DCA_GCE_3_ samples that were prepared under the same curing conditions are compared with that of the DCA_BADGE_2_ in Figure 7a. Here, an overall increase in adhesive strength is observed upon the addition of GCE, with the highest adhesive strength of 11.1 MPa being obtained for the DCA_GCE_2_. With an increase in GCE content, the concentration of C=C radicals grows, resulting in a higher crosslinking density from UV exposure. Consequently, the reduced adhesive strength of DCA_GCE_1_ is likely due to its lower crosslinking density and the presence of unreacted components, even after thermal curing. However, the DCA_GCE_3_ exhibited low adhesive strength despite having a high crosslinking density (as indicated by the gel fraction results). This result can be attributed to the shrinkage of the adhesive network caused by the high crosslinking density, which weakened the interactions at the interface between the adhesive and the substrate [21]. Additionally, the high GCE content of 0.3 moles (i.e., the DCA_GCE_3_) leads to shrinkage, resulting in the formation of a poor network configuration; hence, a reduction in the adhesive strength is observed relative to that of the DCA_GCE_2_. Notably, the DCA_GCE_0_, DCA_GCE_1_, and DCA_GCE_3_ adhesives showed a standard deviation of over 5% across three separate measurements, while the DCA_GCE_2_ adhesive exhibited a reduced deviation of 2%. Moreover, the adhesive strength of DCA_GCE_2_ is similar to that of the DCA_BADGE_2_. This demonstrates that the bonding between the GCE and the polymer network is stabilized and strengthened due to the additional crosslinking in the latter sample [22]. These results suggest the potential use of GCE as an adhesive material in place of the traditional general-purpose resin. With this in mind, other important properties of the GCE are evaluated below.

Figure 7b illustrates the stress–strain curve of DCA adhesive, revealing a decline in stress after reaching maximum strength due to adhesive failure. This behavior is attributed to peeling at the adhesive interface under stress. In contrast, DCA_BADGE_2_ exhibited complete peeling near the maximum strength, indicating that it is relatively more brittle compared to adhesives containing GCE [23]. Additionally, the adhesives with GCE showed higher strain values, and it was observed that the strain increased with higher GCE content.

### 3.4. Thermal Properties

**Figure 8:**
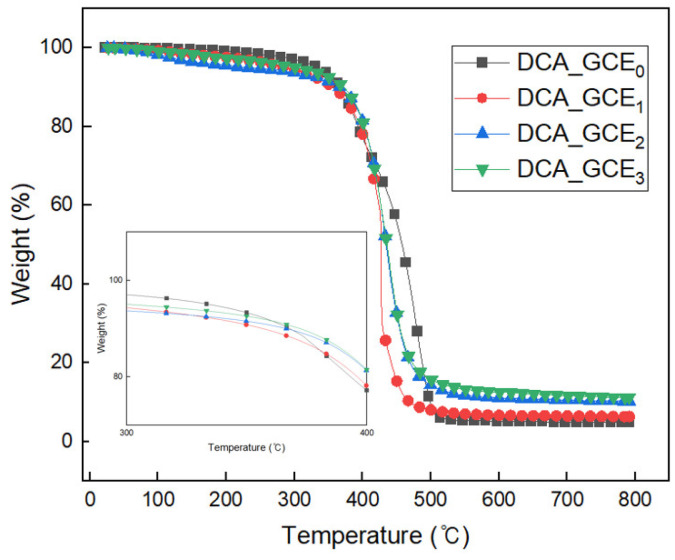
The TGA curves of the various cured samples with and without DCA.

Figure 8 shows the TGA curves for DCA_GCE_0_ and the various GCE-containing DCA samples. The GCE-containing samples display a slight initial weight loss as the temperature rises to 100 °C, which can be attributed to the evaporation of residual solvents. Subsequently, a further decrease begins at around 250 °C due to the decomposition of the soft urethane foam in the GCE-containing samples [24], and this is followed by a rapid decrease at around 300 to 450 °C due to the decomposition of the hard urethane foam, the collapse of the C–O–C bonds, and the decomposition of the copolymer carboxyl group [25]. Notably, the remaining weight at temperatures above 500 °C increases in proportion to the GCE content. These results demonstrate that GCE is effective for improving the thermal resistance characteristics of the DCA.

### 3.5. Surface Properties

The pencil hardness tests of the various DCA samples are presented in Table 2, along with their water and ethylene contact angles and the calculated surface energies. For comparison, the contact angles and surface energy of the commercial polycarbonate (PC) film that was used in the adhesion test are included. Here, the pencil hardness appears to decrease to at least B as the GCE content increases, which is due to an increase in the corresponding urethane foam. The adhesive material obtained from such a decrease in pencil hardness has the advantage of flexibility for application to curved surfaces. Meanwhile, as the GCE content increases, the contact angles rise while the surface energy decreases. This is due to the relatively longer molecular chain of GCE compared to the monomers that form the DCA network without GCE. In general, the interfacial compatibility between an adhesive and a substrate is closely related to the similarity in surface energy values of the two materials. Since plastics such as PC generally have low surface energies, the observed improvement in interfacial adhesion between the PC and the DCA can be attributed to the reduction in surface energy caused by the incorporation of GCE, thereby improving the compatibility between the adhesive and the plastic [26]. Moreover, the greatest improvement in adhesion strength was observed for DCA_GCE_2_, which had the most similar surface energy to that of the PC film.

## 4. Conclusions

The successful preparation of a novel glycidyl carbamate epoxy resin (GCE) was demonstrated herein, with its properties investigated using dual-curing adhesives (DCAs) containing varying GCE contents. Despite the reduction in surface energy with increasing GCE content, the enhanced crosslinking density contributed to the improved adhesion properties, particularly on low-energy surfaces like PC film. This indicates that GCE-based adhesives are effective in bonding to plastic substrates, where both flexibility and thermal stability are required. In addition, the GCE-based adhesives exhibited strong potential for thermal stability and flexibility, making them suitable candidates for high-performance applications. Modifications to the adhesive formulation, such as adjusting comonomer ratios, could further optimize both surface energy and adhesion. These findings highlight GCE as a promising alternative to traditional thermosetting materials, particularly in applications that demand a balance of adhesion strength, flexibility, and thermal resistance.

## Figures and Tables

**Table 1:** The formulations of the different DCAs.

Sample	AA (mol)	TMPTA (mol)	GCE (mol)	BADGE (mol)
DCA_GCE_0_	0.2	0.1	0	0
DCA_GCE_1_	0.2	0.1	0.1	0
DCA_GCE_2_	0.2	0.1	0.2	0
DCA_GCE_3_	0.2	0.1	0.3	0
DCA_BADGE_2_	0.2	0.1	0	0.2

**Table 2:** Pencil hardness, contact angles, and surface energies of different DCA samples. For comparison, the contact angles and surface energy of the PC film that were used in the adhesion test are also shown.

Sample	Pencil Hardness	Contact Angle (°)		Surface Energy (mJ/m^2^)
		Water	Ethylene Glycol	
DCA_GCE_0_	H	37.24	22.61	54.45
DCA_GCE_1_	HB	69.78	41.27	45.70
DCA_GCE_2_	HB	85.41	58.25	32.97
DCA_GCE_3_	B	101.22	70.94	31.84
DCA_BADGE_2_	H	69.77	40.14	45.84
PC film	-	79.35	56.08	35.74

## Data Availability

Data are contained within the article.
